# Modulation of Prostanoids Profile and Counter-Regulation of SDF-1α/CXCR4 and VIP/VPAC2 Expression by Sitagliptin in Non-Diabetic Rat Model of Hepatic Ischemia-Reperfusion Injury

**DOI:** 10.3390/ijms222313155

**Published:** 2021-12-05

**Authors:** Małgorzata Krzystek-Korpacka, Mariusz G. Fleszar, Paulina Fortuna, Kinga Gostomska-Pampuch, Łukasz Lewandowski, Tomasz Piasecki, Bogna Kosyk, Adam Szeląg, Małgorzata Trocha

**Affiliations:** 1 Department of Biochemistry and Immunochemistry, Wroclaw Medical University, 50-368 Wrocław, Poland; mariusz.fleszar@umw.edu.pl (M.G.F.); paulina.fortuna@umw.edu.pl (P.F.); kinga.gostomska-pampuch@umw.edu.pl (K.G.-P.); lukasz.lewandowski@umw.edu.pl (Ł.L.); 2 Department of Epizootiology and Clinic of Bird and Exotic Animals, Wroclaw University of Environmental and Life Sciences, 50-366 Wrocław, Poland; tomasz.piasecki@upwr.edu.pl; 3 Institute of Soil Science and Environmental Protection, Wroclaw University of Environmental and Life Sciences, 50-375 Wroclaw, Poland; bogna.kosyk@upwr.edu.pl; 4 Department of Pharmacology, Wroclaw Medical University, 50-345 Wrocław, Poland; adam.szelag@umw.edu.pl

**Keywords:** drug repurposing, incretins, prostaglandins, vasoactive intestinal peptide (VIP), stromal-derived factor 1α (SDF-1α), glucagon-like peptide 1 (GLP-1), dipeptidyl peptidase IV (DPP4), gliptins, liver transplantation, hepatoprotection

## Abstract

Molecular mechanisms underlying the beneficial effect of sitagliptin repurposed for hepatic ischemia-reperfusion injury (IRI) are poorly understood. We aimed to evaluate the impact of IRI and sitagliptin on the hepatic profile of eicosanoids (LC-MS/MS) and expression/concentration (RTqPCR/ELISA) of GLP-1/GLP-1R, SDF-1α/CXCR4 and VIP/VPAC1, VPAC2, and PAC1 in 36 rats. Animals were divided into four groups and subjected to ischemia (60 min) and reperfusion (24 h) with or without pretreatment with sitagliptin (5 mg/kg) (IR and SIR) or sham-operated with or without sitagliptin pretreatment (controls and sitagliptin). PGI_2_, PGE_2_, and 13,14-dihydro-PGE_1_ were significantly upregulated in IR but not SIR, while sitagliptin upregulated PGD_2_ and 15-deoxy-12,14-PGJ_2_. IR and sitagliptin non-significantly upregulated GLP-1 while *Glp1r* expression was borderline detectable. VIP concentration and *Vpac2* expression were downregulated in IR but not SIR, while *Vpac1* was significantly downregulated solely in SIR. IRI upregulated both CXCR4 expression and concentration, and sitagliptin pretreatment abrogated receptor overexpression and downregulated *Sdf1*. In conclusion, hepatic IRI is accompanied by an elevation in proinflammatory prostanoids and overexpression of CXCR4, combined with downregulation of VIP/VPAC2. Beneficial effects of sitagliptin during hepatic IRI might be mediated by drug-induced normalization of proinflammatory prostanoids and upregulation of PGD_2_ and by concomitant downregulation of SDF-1α/CXCR4 and reinstating VIP/VCAP2 signaling.

## 1. Introduction

Liver transplantation is a life-saving procedure for patients with end-stage liver disease, the incidence of which is rising along with the increasing prevalence of its risk factors such as alcoholic and non-alcoholic steatohepatitis, viral infections, and cancer. While procedure frequency is constantly increasing as well, the number of patients requiring transplant exceeds the organ availability. Transplantation is associated with a significant risk of graft rejection, and an ischemia/reperfusion (IR) injury during transplantation is one of the key contributors. Moreover, the IR has a negative impact on the functioning of transplanted organs [1,2].

The IR injury is a complex phenomenon, and its pathology is not fully elucidated. A better understanding of its cellular and molecular mechanisms is needed to develop strategies protecting organs during and after transplantation [1,2]. Ischemia causes metabolic imbalance characterized by acidosis and ATP depletion, inducing apoptosis. Unfavorable changes in this phase intensify in the course of reperfusion. Activation of Kupffer cells and infiltration with T lymphocytes during the early phase of reperfusion and accumulation of macrophages and neutrophils during the late phase leads to the release of a plethora of inflammatory and immune mediators and generation of molecule-damaging reactive oxygen and nitrogen species [2].

IR event is accompanied by a release of vast amounts of arachidonic acid, an eicosanoid, from membrane phospholipids by phospholipase A2 (PLA2). Arachidonic acid is further converted to PGH_2_ by two isoforms of cyclooxygenase (COX), a constitutive COX1 and an inducible COX2. Subsequently, PGH_2_ is metabolized to prostacyclin PGI_2_, prostaglandins (PGs) D_2_, E_2,_ and F_2_, and thromboxane (TX) A_2_, referred to as prostanoids. The 5-lipoxygenase, in turn, converts arachidonic acid into leukotriene (LT) A_4_. Less abundant dihomo-γ-linolenic acid, also metabolized by COX enzymes, becomes a precursor of series 1 of prostanoids, e.g., PGE_1_ [3]. Eicosanoids are frequently their own functional antagonists and display diverse biological activities, depending on their source, cell type, partner downstream receptors, and the context. Simplifying, COX1-derived prostanoids are mostly involved in housekeeping functions, while COX2-derived mediators are engaged in immune and inflammatory responses [4].

Hepatic IR is associated with the upregulated activity of PLA2 and COX2 and the accumulation of PGE_2_, produced mainly by hepatocytes and endothelial and Kupffer cells (reviewed in the work of [5]). However, the role of PGE_2_, a main proinflammatory prostanoid [3,4], in hepatic IR remains controversial. On the one hand, COX depletion [6] or inhibition by aspirin [7] protects the liver during IR. Consistently, inhibition of upstream PLA2 by dexamethasone [7,8] or downstream inducible PGE_2_ synthase (mPGES-1) [9] confers protection as well. On the other, mesenchymal stem cell-derived PGE_2_ [10] or PGE_2_ signaling via EP_4_ receptor [11] are claimed to exert hepatoprotective effects during liver injury.

Apart from steroidal and non-steroidal anti-inflammatory drugs, the efficacy of other well-established pharmaceuticals in alleviating organ IR injury is currently being investigated. Among these, gliptins (dipeptidyl peptidase-4 (DPP-4) inhibitors and primarily antidiabetics) arouse a growing interest. Gliptins, including their protagonist, sitagliptin, have been shown to exert cytoprotective effects in animal models of hepatic [12,13,14,15,16,17,18], cardiac [19,20], renal [21,22,23], cerebral [24], testicular [25], and intestinal [26,27] IR injury. Although anti-inflammatory, antioxidant, and antiapoptotic properties of gliptins seem to be implicated, the underlying molecular mechanisms are to be explained.

This work was designed to evaluate the effect of IR injury and sitagliptin on rat liver profile of eicosanoids: 6-ketoPGF_1α_, a stable metabolite of PGI_2_; PGE_2_; PGD_2_; PGF_2_; 15-deoxy-12,14-PGJ_2_, a PGD_2_ metabolite; 13,14-dihydro-PGE_1_, a PGE_1_ metabolite; TXB_2_, a stable metabolite of TXA_2_; and LTB_4_, a stable LTA_4_ metabolite, in the wide context of inflammatory mediators and oxidative, nitrosative, and halogenative stress markers. It was also aimed at analyzing the IR and sitagliptin impact on the expression and/or concentration of potentially relevant substrates of DPP-4 and their receptors, namely, SDF1/CXCR4, GLP1/GLP1R and VIP/VPAC1 and VPAC2, and PAC1.

## 2. Results

### 2.1. A Rat Model of IR and Its Validation

**Figure 1:**
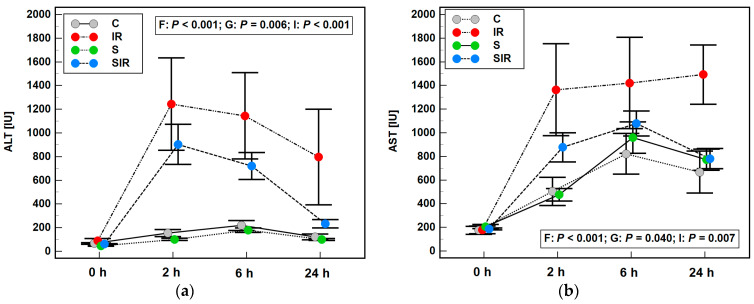
Validation of hepatic ischemia-reperfusion model—the effect of injury on the dynamics of liver enzymes: (**a**) alanine aminotransferase (ALT); (**b**) aspartate aminotransferase. Data are presented as mean ± SEM and were analyzed using repeated-measures ANOVA. F, the effect of factor (time of blood sampling after IR); G, the effect of group (C, IR, S, SIR); I, the effect of factor × group interaction; C, control sham-operated animals; IR, animals subjected to ischemia-reperfusion; S, sham-operated animals pretreated with sitagliptin; SIR, animals subjected to ischemia-reperfusion pretreated with sitagliptin.

A rat IR model has previously been established [15,16], in which 36 animals were randomized into four groups: sham-operated (controls; *n* = 9), sham-operated following pretreatment with sitagliptin (5 mg/kg p.o.) (sitagliptin; *n* = 8), subjected to IR procedure (IR; *n* = 9), and subjected to IR procedure following pretreatment with sitagliptin (SIR; *n* = 10). It was validated by an elevation in the activity of alanine (ALT) and aspartate (AST) aminotransferases following injury, less pronounced in SIR than the IR group. The repeated-measures ANOVA indicated significant effect of factor (time of blood collection: 0, 2, 6, and 24 h), group (control, IR, sitagliptin, SIR) as well as factor × group interaction on ALT and AST dynamics (Figure 1). In addition, histopathological analysis indicated slight necrotic changes as well as neutrophil infiltration in IR and SIR animals and a significantly higher degree of steatosis in animals pretreated with sitagliptin [16] (Supplementary Figures S1–S4).

### 2.2. Effect of IR Injury and Sitagliptin on Liver Profile of Eicosanoids

The profile of eicosanoids was determined using liquid chromatography-tandem mass spectrometry (LC-MS/MS).

The concentrations of 6-ketoPGF_1α_ (PGI_2_), PGE_2_, and 13,14-dihydro-PGE_1_ in the liver were significantly upregulated in the IR group as compared to controls and IR animals pretreated with sitagliptin (SIR group). In addition, 6-ketoPGF_1α_ (PGI_2_), but not PGE_2_ and 13,14-dihydro-PGE_1_, were also upregulated in the sitagliptin group as compared to controls and SIR animals (Table 1).

Sitagliptin significantly upregulated PGD_2_, which was higher than in controls, IR, and SIR animals. It also upregulated 15-deoxy-12,14-PGJ_2_, which was elevated in sitagliptin and SIR groups as compared to controls and IR animals (Table 1).

Liver concentrations of PGF_2_, TBX_2,_ and LTB_4_ were affected neither by IR nor sitagliptin. The PGI_2_-to-TXA_4_ ratio, calculated based on their stable metabolites (6-ketoPGF_1α_/TXB_2_), was higher in IR as compared to controls and SIR, but sitagliptin alone also elevated the ratio as compared to control animals (Table 1).

### 2.3. Effect of IR Injury and Sitagliptin on Liver Expression of CXCR4/SDF1

The expression and concentration of SDF1 cytokine and CXCR4, its receptor, were determined using reversely transcribed quantitative polymerase chain reaction (RTqPCR) and immunoenzymatic assays (ELISA).

**Figure 2:**
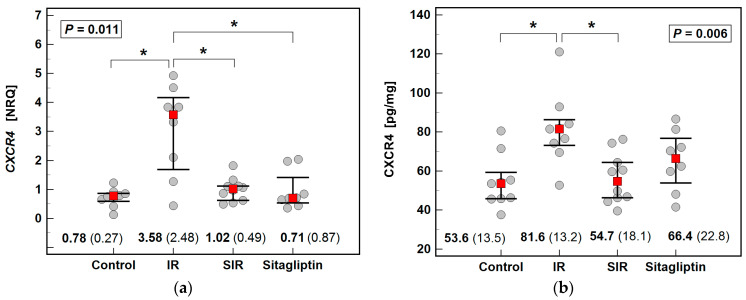

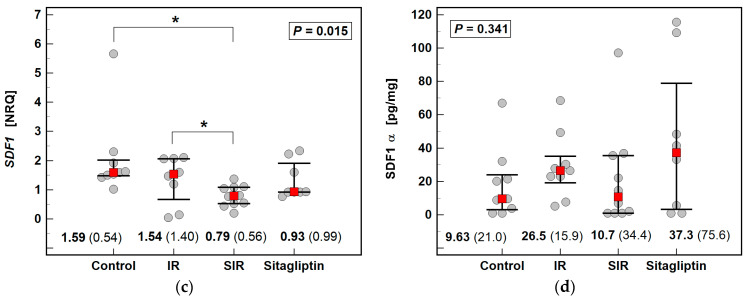
Effect of IR injury and sitagliptin on CXCR4/SDF1 axis in the liver: (**a**) *Cxcr4* expression; (**b**) CXCR4 concentration; (**c**) *Sdf1* expression; (**d**) SDF1α concentration. Data were analyzed using the Kruskal–Wallis *H* test and are presented as medians with IQR (red squares with whiskers and numbers below dot-plots). Significant (*p* < 0.05) differences between groups, identified in *post-hoc* analysis (Conover test), are indicated by connectors with * symbol. NRQ, normalized relative quantity; IR, ischemia-reperfusion; SIR, sitagliptin pretreatment and ischemia-reperfusion; IQR, interquartile range.

The IR significantly upregulated CXCR4, both at mRNA and protein level, and pretreatment with sitagliptin downregulated its expression and concentration. Regarding the CXCR4 ligand SDF1, its expression (*Sdf1*) was significantly lower in the SIR group as compared to IR and controls. Neither IR nor sitagliptin had a significant impact on SDF1α (Figure 2).

### 2.4. Effect of IR Injury and Sitagliptin on Liver Expression of PAC1, VPAC1, VPAC2/VIP

**Figure 3:**
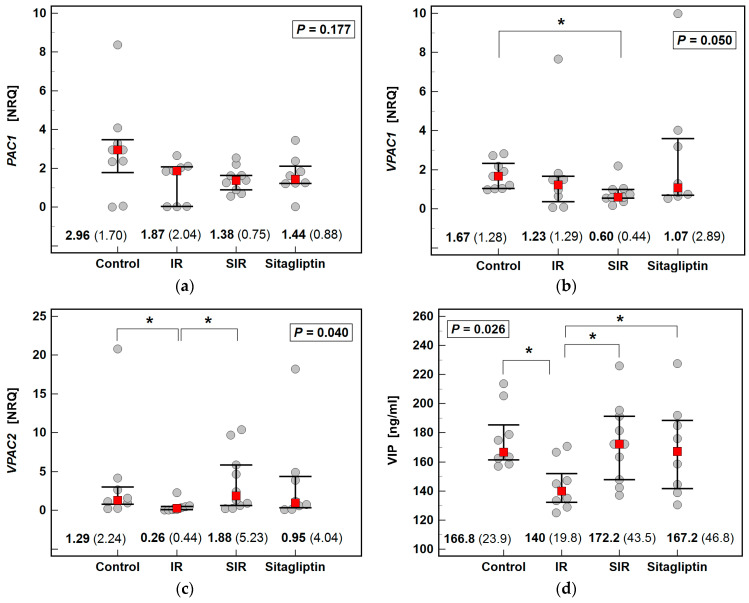
Effect of IR injury and sitagliptin on PAC1, VPAC1, VPAC2/VIP axis in the liver: (**a**) *Pac1* expression; (**b**) *Vpac1* expression; (**c**) *Vpac2* expression; (**d**) VIP concentration. Data were analyzed using the Kruskal–Wallis *H* test and are presented as medians with IQR (red squares with whiskers and numbers below dot-plots). Significant (*p* < 0.05) differences between groups, identified in *post-hoc* analysis (Conover test), are indicated by connectors with * symbol. NRQ, normalized relative quantity; IR, ischemia-reperfusion; SIR, sitagliptin pretreatment and ischemia-reperfusion; IQR, interquartile range.

The IR significantly downregulated the expression of *Vpac2* and concentration of VIP while pretreatment with sitagliptin prevented the downregulation of both the receptor expression and ligand concentration. The IR non-significantly downregulated *Pac1* and *Vpac1*, the expression of which was more markedly downregulated in SIR animals, significantly so in the case of *Vpac1* expression (Figure 3).

### 2.5. Effect of IR Injury and Sitagliptin on Liver Expression of GLP1R/GLP1

**Figure 4:**
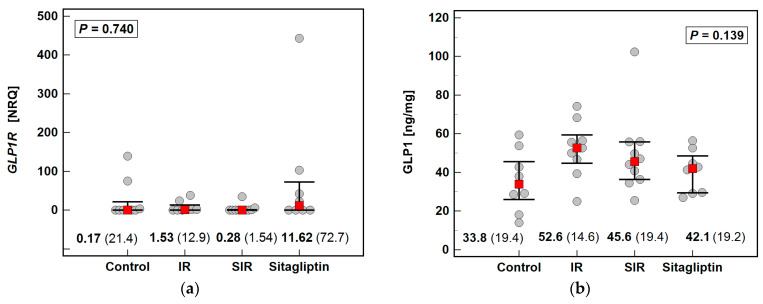
Effect of IR injury and sitagliptin on GLP1R/GLP1 axis in the liver: (**a**) *Glp1r* expression; (**b**) GLP1 concentration. Data were analyzed using the Kruskal–Wallis *H* test and are presented as medians with IQR (red squares with whiskers and numbers below dot-plots). NRQ, normalized relative quantity; IR, ischemia-reperfusion; SIR, sitagliptin pretreatment and ischemia-reperfusion; IQR, interquartile range.

Neither IR nor sitagliptin had a significant impact on *Glp1r* expression and GLP1 concentration in the liver (Figure 4).

### 2.6. Relationship between Eicosanoids, DPP4 Ligands, and Their Receptors, and Mediators of Inflammation and Oxidative, Nitrosative, and Halogenative Stress (Univariate Analysis)

Liver eicosanoids as well as investigated DPP4 ligands and their receptors were related to the expression or concentration of mediators of inflammation and oxidative, nitrosative and halogenative stress: IL-1β, IL-10, IFNγ, VEGF-A, MIP-2, TNFα, *Il6*, *Mmp1*, *Nampt*, *Tnfa*, *Nox1*, *Nox2*, *Nox4*, *Mdk*, *Ptn*, 3-nitrotyrosine (NT), and 3-bromotyrosine (BT).

#### 2.6.1. Eicosanoids

In univariate analysis, 6-ketoPGF_1α_ (PGI_2_) correlated with the concentration of 13,14-dihydro-PGE_1_ (*r* = 0.36, *p* = 0.031), LBT_4_ (*r* = 0.35, *p* = 0.035), PGD_2_ (*r* = 0.45, *p* = 0.006), PGE_2_ (*r* = 0.61, *p* < 0.001), CXCR4 (*r* = 0.55, *p* < 0.001), 3-NT (*r* = 0.38, *p* = 0.021), SDF1α (*r* = 0.40, *p* = 0.016), VIP (*ρ* = −0.51, *p* = 0.002), and IL-1β (*r* = 0.33, *p* = 0.049) and the expression of *Nox4* (*r* = −0.67, *p* < 0.001), *Ptn* (*r* = −0.38, *p* = 0.023), *Sdf1* (*r* = −0.44, *p* = 0.009), *Vpac2* (*r* = −0.38, *p* = 0.026), *Vpac1* (*r* = −0.34, *p* = 0.045), and *Cxcr4* (*r* = 0.48, *p* = 0.003).

Apart from 6-ketoPGF_1α_ (PGI_2_), the concentration of PGE_2_ corelated with LBT_4_ (*r* = 0.64, *p* < 0.001), PGD_2_ (*r* = 0.55, *p* < 0.001), PGF_2_ (*r* = 0.69, *p* < 0.001), CXCR_4_ (*r* = 0.34, *p* = 0.043), SDF1α (*r* = 0.54, *p* < 0.001), and VIP (*r* = −0.42, *p* = 0.010) and the expression of *Nox4* (*r* = −0.42, *p* = 0.012) and *Cxcr4* (*r* = 0.43, *p* = 0.011).

Apart from PGE_2_, the concentration of PGF_2_ correlated with these of LTB_4_ (*r* = 0.42, *p* = 0.010), TXB_2_ (*r* = 0.59, *p* < 0.001), PGD_2_ (*r* = 0.36, *p* = 0.033), and SDF1α (*r* = 0.41, *p* = 0.012).

In addition to 6-ketoPGF_1α_ (PGI_2_), PGE_2_, and PGF_2_, the concentration of PGD_2_ corelated with 15-deoxy-12,14-PGJ_2_ (*r* = 0.56, *p* < 0.001), LBT_4_ (*r* = 0.59, *p* < 0.001), and SDF1α (*r* = 0.59, *p* < 0.001).

Apart from PGD_2_, the concentration of 15-deoxy-12,14-PGJ_2_ correlated with that of LTB_4_ (*r* = 0.44, *p* = 0.009).

The 13,14-dihydro-PGE_1_ correlated solely with 6-ketoPGF_1α_ (PGI_2_).

Apart from PGF_2_, the concentration of TXB_2_ correlated with the expression of *Nox2* (*r* = −0.36, *p* = 0.035).

In addition to prostanoids: 15-deoxy-12,14-PGJ_2_, PGI_2_, PGD_2_, PGE_2,_ and PGF_2_, the concentration of LBT_4_ correlated with that of SDF1α (*r* = 0.59, *p* < 0.001).

#### 2.6.2. CXCR4/SDF1

The concentration of CXCR4 correlated with that of PGE_2_ and 6-ketoPGF_1α_ (PGI_2_) and the expression of *Cxcr4* (*r* = 0.48, *p* = 0.004) and *Vpac2* (*r* = −0.40, *p* = 0.019). The receptor expression (*Cxcr4*) correlated with the expression of *Nox4* (*r* = −0.34, *p* = 0.044) and *Il6* (*r* = 0.40, *p* = 0.018) and GLP1 concentration (*r* = 0.34, *p* = 0.046), in addition to CXCR4 protein and the concentrations of 6-ketoPGF_1α_ (PGI_2_) and PGE_2_.

The concentration of SDF1α correlated with that of 6-ketoPGF_1α_ (PGI_2_), PGD_2_, PGE_2_, PGF_2_, and LTB_4_. Ligand expression (*Sdf1*), in turn, correlated with the concentration of 6-ketoPGF_1α_ (PGI_2_) and IL-1β (*r* = −0.36, *p* = 0.034) and the expression of *Mdk* (*r* = 0.41, *p* = 0.014), *Ptn* (*r* = 0.70, *p* < 0.001), *Nox1* (*r* = 0.38, *p* = 0.027), *Nox4* (*r* = 0.53, *p* = 0.001), *Vpac1* (*r* = 0.79, *p* < 0.001), *Mmp1* (*r* = 0.51, *p* = 0.002), and *Nampt* (*r* = 0.68, *p* < 0.001).

#### 2.6.3. PAC1, VPAC1, VPAC2/VIP

The expression of *Vpac1* correlated with 6-ketoPGF_1α_ (PGI_2_) concentration and the expression of *Sdf1, Glp1r* (*r* = 0.69, *p* < 0.001), *Mdk* (*r* = 0.79, *p* < 0.001), *Ptn* (*r* = 0.87, *p* < 0.001), *Nox1* (*r* = 0.73, *p* < 0.001), *Nox2* (*r* = −0.43, *p* = 0.009), *Mmp1* (*r* = 0.80, *p* < 0.001), *Nampt* (*r* = 0.53, *p* = 0.001), and *Tnfa* (*r* = 0.37, *p* = 0.029).

The expression of *Vpac2* correlated with liver concentrations of CXCR4, 6-ketoPGF_1α_ (PGI_2_), and GLP1 (*r* = −0.37, *p* = 0.030), whereas the expression of *Pac1* did not show any significant correlation.

The concentration of VIP correlated with 6-ketoPGF_1α_ (PGI_2_), PGE_2_, and 3-BT (*r* = −0.33, *p* = 0.049) and the expression of *Nox4* (*r* = 0.40, *p* = 0.017).

#### 2.6.4. GLP1R/GLP1

The expression of *Glp1r* correlated with these of *Vpac1, Mdk* (*r* = 0.84, *p* < 0.001), *Ptn* (*r* = 0.68, *p* < 0.001), *Nox1* (*r* = 0.90, *p* < 0.001), *Nox2* (*r* = −0.43, *p* = 0.011), *Il6* (*r* = 0.47, *p* = 0.004), *Tnfa* (*r* = 0.71, *p* < 0.001), and *Mmp1* (*r* = 0.83, *p* < 0.001).

GLP1 correlated with IFNγ (*r* = −0.37, *p* = 0.025) in addition to *Cxcr4* and *Vpac2* expression.

### 2.7. Independent Predictors of Liver Eicosanoids and DPP4 Ligands and Their Receptors (Multivariate Analysis)

Multiple linear regression analysis (stepwise method) was conducted to discern independent predictors of variability in the concentration of individual eicosanoids as well as concentration/expression of investigated DPP4 ligands and their receptors.

#### 2.7.1. Independent Predictors of Liver Eicosanoids

Of the variables found significantly associated in univariate analysis, 13,14-dihydro-PGE_1_, PGE_2_, 3-NT, VIP, IL-1β, *Cxcr4,* and *Nox4* were independent predictors of 6-ketoPGF_1α_ (PGI_2_), explaining 83% variability in its concentration.

6-ketoPGF_1α_ (PGI_2_), PGF_2,_ and LTB_4_ were independent predictors of PGE_2_, explaining 74% of variability, and PGE_2_ and TXB_2_ were independent predictors of PGF_2_, explaining 61% in its variability. PGF_2_ was independently associated with TXB_2_, explaining 28% in its variability, and 15-deoxy-12,14-PGJ_2_, PGE_2,_ and SDF1α were independent predictors of LTB_4_, explaining 62% in its variability.

The IR was a sole predictor of 13,14-dihydro-PGE_1_, which explained 16% in concentration variability. Sitagliptin was an independent predictor of 15-deoxy-12,14-PGJ_2_, which, together with PGD_2_, explained 45% in concentration variability and of PGD_2_, for which it explained 73% of variability together with 15-deoxy-12,14-PGJ_2_, PGE_2_, and SDF1α (Table 2).

#### 2.7.2. Independent Predictors of Liver Expression of DPP4 Ligands and Their Receptors

The IR was an independent predictor of the concentration of CXCR4, explaining 39% of its variability together with 6-ketoPGF_1α_ (PGI_2_) and the concentration of VIP, explaining 39% of its variability together with PGE_2_ and 3-BT. Together with IFNγ, it also explained 32% of the variability in GLP1 concentration. The IR was a sole predictor of *Cxcr4* and *Vpac2* expression, explaining, respectively, 38% and 23% in their variability (Table 3).

The variability in SDF1α concentration was explained in 44% by LTB_4_ and PGD_2_, while that in *Sdf1* expression in 84% by changes in expression of *Vpac1*, *Mdk*, *Nox4*, and *Nampt* (Table 3).

The variability in the expression of the *Vpac1* receptor was explained in 91% by changes in *Mdk*, *Nox2*, *Ptn*, and *Sdf1* expression and this in *Glp1r* in 83% by changes in *Nox1* and *Nox2* expression (Table 3).

## 3. Discussion

Deciphering molecular mechanisms of hepatic IR injury is a prerequisite to developing successful treatment strategies that would improve patients’ survival by reducing rates of graft rejection and improving the function of transplanted organs [2]. The ATP depletion in sinusoidal endothelial cells and hepatocytes caused by ischemia leads to the generation of reactive oxygen species (ROS), cell death, and release of alarmins, activating resident neutrophils and Kupfer cells. Released ROS and cytokines damage macromolecules and recruit circulating immune cells as well as hepatic stellate cells, engaging them in self-perpetuating inflammation and oxidative stress [2]. Therefore, targeting oxidative stress, inflammation, and preventing apoptosis are viewed as promising therapeutic options.

Prostanoids are potent mediators of immune and inflammatory responses. Under physiological conditions, prostanoids maintain homeostasis and are hepatoprotective. However, both their depletion and excess, occurring as a consequence of COX-2 activation in response to insult, might be detrimental [28]. In the IR settings accompanied by reduction in prostanoids’ concentration, a treatment with prostaglandins seems to be beneficial due to improved liver hemodynamics and survival of sinusoidal epithelial cells and reduced generation of ROS, leukocyte migration, and platelet aggregation (reviewed in the work of [29]). However, the efficacy of none of the tested prostaglandins, that is, PGE_1_, PGE_2,_ or PGI_2_, has been confirmed in clinical trials [2]. Moreover, COX2 has been ascribed both a protective [30] and a critical enabling role in hepatic IR injury [6]. Adding to the confusion, COX2 inhibition [7] or inhibition of any other enzyme involved in the synthesis of eicosanoids [7,8,9] have proved beneficial.

In the IR injury model investigated here, hepatic concentrations of 13,14-dihydro-PGE_1_, 6-keto-PGF_1α_, and PGE_2_ were elevated in IR as compared to sham-operated animals while these of PGD_2_, its metabolite 15-deoxy-12,14-PGJ_2_, PGF_2_, TXB_4,_ and LTB_4_ were not significantly altered. Similar IR-induced alterations in the profile of prostanoids were reported in the heart by Qiu et al. [31] and demonstrated to contribute to cardiac apoptosis. The authors have observed the upregulation of 6-keto-PGF_1α_, PGE_2_, PGD_2_ and no change in TXB_2_ and PGF_2α_ (13,14-dihydro-PGE_1_, 15-deoxy-12,14-PGJ_2_, and LTB_4_ were not determined). The upregulation of PGI_2_ and an elevation of PGI_2_/TXA_2_ ratio have also characterized rat hippocamps in the course of cerebral IR injury but have been interpreted as a physiological mechanism aimed at neuroprotection [32].

The multivariate analysis conducted in the present study demonstrated that IR was an independent predictor of solely 13,14-dihydro-PGE_1_. However, 13,14-dihydro-PGE_1_ was, in turn, an independent predictor of 6-ketoPGF_1α_ (PGI_2_), which explained variability in PGE_2_, a contributor to the variability in PGF_2_, PGI_2_, PGD_2_, and LTB_4_, stressing strong interrelationships between all studied eicosanoids. The stimulatory effect of inflammation and oxidative stress on their synthesis was indicated by the inclusion of IL-1β and 3-NT, an oxidative/nitrosative stress marker, in the regression models predicting prostacyclin 6-ketoPGF_1α_ (PGI_2_) concentration as well as by including SDF-1α or CXCR4 in the regression models explaining the variability in 6-ketoPGF_1α_ (PGI_2_), PGD_2_, and LTB_4_ concentration.

There is an increasing interest in repurposing the well-established drugs with already known toxicity, pharmacokinetics, and pharmacodynamics [33], including repositioning gliptins for IR injury. The contribution of antioxidant, anti-inflammatory, and antiapoptotic properties of sitagliptin to liver protection during IR injury has been well documented [13,14,15,16,17,18]. However, the drug effect on the liver profile of eicosanoids during IR has not been investigated, although sitagliptin has been demonstrated to reduce both PLA2 and COX2 concentration in various clinical and experimental settings [34,35]. Consistently, sitagliptin pretreatment of IR animals prevented significant elevation of 6-ketoPGF_1α_ (PGI_2_), PGE_2_, and 13,14-dihydro-PGE_1_ (PGE_1_) and reduced 6-ketoPGF_1α_ (PGI_2_)/TXB_2_ (TXA_2_) ratio, elevated in IR. Interestingly, sitagliptin in control sham-operated animals had an opposite effect; it elevated the expression of 6-ketoPGF_1α_ (PGI_2_) and 6-ketoPGF_1α_ (PGI_2_)/TXB_2_ (TXA_2_) ratio, indicating that the biological effect of the drug is context-dependent.

Sitagliptin, but not IR, was responsible for an elevation of PGD_2_ and 15-deoxy-12,14-PGJ_2_ (PGD_2_), significant solely in sitagliptin-pretreated control animals (PGD_2_) or in both control and IR animals in (PGD_2_ metabolite). The unfavorable proinflammatory character of PGD_2_ is manifested mostly in the lung and in allergic and autoimmune diseases, while in the liver, PGD_2_ has only recently been shown to alleviate the injury during IR [36]. Specifically, PGD_2_ metabolite 15-deoxy-Δ12,14-prostaglandin J_2_ exerted hepatoprotective effects by alleviating oxidative stress via activation of Nrf2, reducing systemic inflammation and liver infiltration with macrophages, decreasing apoptotic rates, and by downregulating an expression of beclin-1 and LC3, autophagy markers. Activation of Nrf2 signaling in IR liver in response to sitagliptin without exploring the mechanism has previously been noted by Abdel-Gaber et al. [14]. Sitagliptin has also been shown to alleviate hepatic fibrosis by mechanisms involving the enhancement of Nrf2 expression accompanied by the downregulation of NFκB [37]. Therefore, our observation on sitagliptin-mediated upregulation of PGD_2_ and its metabolite in the context of its ability to induce Nrf2 signaling might shed some light on the molecular mechanism involved. PGD_2_ might also mediate, recently reported [38], sitagliptin-induced downregulation of LC3 and beclin-1 in endothelial cells.

The number of studies on gliptins as attenuators of IR injury, which would explore possible mechanisms in addition to documenting drug anti-inflammatory and antioxidant properties, is limited. Sitagliptin, a competitive inhibitor of DPP4 also found to downregulate the enzyme expression [18], exerts its antidiabetic activity by preserving GLP-1, a peptide hormone synthesized by gut L cells [39]. GLP-1 and its analogs display antiapoptotic, anti-inflammatory, and antioxidant activities conferring protection during myocardial [40] and cerebral [41] IR. Consistently, sitagliptin attenuated intestinal IR injury by inducing GLP-1/GLP-1R signaling [26] and renal IR injury by upregulating GLP-1 secretion and local GLP-1R expression [23,42]. In the liver, GLP-1/GLP-1R has been speculated to mediate the upregulation of Nrf2 expression and subsequent elevation in heme oxygenase-1 [14]. Though, neither GLP-1R nor GLP-1 status in IR liver pretreated with sitagliptin has been investigated by the authors [14]. The speculation was based on, reported elsewhere [43], hepatoprotection conferred by activation of hepatic GLP-1R by receptor agonists. Cytoprotective effects of GLP-1 agonists have also been demonstrated in ischemic stroke [44] and myocardial infarct [45]. However, contrary to other organs, the presence of GLP-1R in the liver is controversial [46,47]. In the present study, GLP-1R mRNA expression was undetectable or borderline detectable in most of the examined livers, with six out of nine measurable cases (Cq < 40) present in sitagliptin-treated animals. The GLP-1 concentration in the liver did not differ significantly between analyzed groups, although a non-significant peptide upregulation in IR animals could be noted. Still, IR was an independent predictor of GLP-1, regardless of sitagliptin pretreatment, exerting a positive effect on hormone concentration. The local concentration of IFNγ, in turn, was a negative predictor of hepatic GLP-1, corroborating previous observations on an inverse relationship between the hormone and IFNγ signaling (reviewed in the work of [34]) and IFNγ involvement in hepatic IR injury [48].

To elucidate potential mechanisms of sitagliptin action in hepatic IR injury, we examined the drug effect on other potential DPP4 substrates, namely VIP and SDFα, and on their receptors. The VIP is a ubiquitous peptide hormone with anti-inflammatory and antioxidant properties, employing mainly VCAP1 and VCAP2 receptors. It can also interact with PACAP receptor (PAC1), although with lower affinity [49]. The VIP has been demonstrated to attenuate IR injury in mice liver [50] and rat lungs [51] and to protect rat kidneys from hemorrhagic ischemia and retransfusion [52]. Specifically, VIP has reduced liver infiltration with macrophages and neutrophils, increased concentration of anti-inflammatory IL-10, and decreased hepatocyte apoptotic rates while downregulating the expression of macrophage-derived TNFα, IL-6, and IL-12 [50]. However, whereas Ji et al. [50] observed an elevation in hepatic VIP mRNA expression in response to IR, our results indicate the downregulation of peptide concentration, which was avoided by sitagliptin preconditioning. Our finding is consistent with accelerated peptide degradation owing to upregulated DPP4 expression in response to hypoxia (reviewed in the work of [53]). Moreover, it also agrees well with an observation made in lung IR injury on the DPP4 inhibition upregulating both VIP concentration and expression [51]. Supporting an inverse relation between VIP and IR injury, peptide concentration in the present study was inversely related to both inflammation and oxidative stress. PGE_2_ and 3-BT concentrations were negative independent predictors of VIP concentration in addition to IR. In turn, VIP was an independent negative predictor of 6-ketoPGF1α, a stable metabolite of PGI_2_. The IR downregulated, and sitagliptin preconditioning also upregulated the expression of *Vcap2*, while *Vcap1* expression was lower in all groups as compared to controls, significantly so in SIR. The IR was a sole, negative predictor of *Vcap2* expression while that of *Vcap1* was independently associated with oxidative stress marker *Nox2* as a negative predictor and with *Ptn*, *Mdk*, and *Sdf1* as positive predictors. Pleiotrophin (*Ptn*) and midkine (*Mdk*) are closely related to heparin-binding growth factors found to support liver regeneration [54] and protect against metal-induced liver toxicity [55] but being oppositely regulated in hepatic IR [17]. Moreover, pleiotrophin has been shown to play a cytoprotective role in brain and heart IR [56,57]. Midkine role during IR injury seems to depend on the affected organ as the cytokine exerted antiapoptotic, thus protective activity in the heart [58] but contributed to organ damage in the renal and hindlimb models of ischemia by promoting macrophage and neutrophil infiltration [59,60].

Instead of activation of GLP-1/GLP-1R, the upregulated Nrf2 expression in the liver subjected to IR injury observed by Abdel-Gaber et al. [14] might be attributed to increased stability of SDF-1α. In lung IR injury models, SDF-1α activates Nrf2 by triggering MAPK and PI3K/Akt pathways, the latter being also involved in SDF1α-mediated interference with NFκB signaling (reviewed in the work of [61]). However, as demonstrated here, hepatic IR had no effect on *Sdf1* expression, and it elevated SDF-1α concentration only non-significantly. This observation opposes the notion of increased expression of SDF-1α upon acute and chronic liver injury, where the cytokine can contribute to both liver regeneration and fibrosis. Those contradictory roles of SDF-1α are believed to be executed by signaling via different receptors: pro-regenerative CXCR7 or profibrotic CXCR4 (reviewed in the work of [62]). Nonetheless, a known stimulatory effect of hypoxia and reactive oxygen species on SDF1α and CXCR4 [63] was, in the case of *Sdf1*, reflected by *Nampt* and *Nox4* being independent positive predictors of cytokine expression. In the case of CXCR4, receptor expression and concentration were significantly upregulated in IR animals, and the IR was an independent predictor of both *Cxcr4* and CXCR4. Receptor overexpression has been detrimental in animal models of renal [64] and cardiac IR [65], in which it induces senescence in progenitor epithelial cells [64] or recruited inflammatory cells and increased production of inflammatory cytokines while inducing apoptosis in cardiomyocytes [65]. Antioxidants [66] and CXCR4 inhibitors [67] have been shown to prevent stress-induced receptor upregulation. In the liver, plerixafor, a specific receptor inhibitor, improves recovery of the liver following IR injury by restoring the proliferative potential of hepatocytes, hampered by CXCR4 signaling [67]. Inhibiting CXCR4 has proven effective also in attenuating oxidative stress-induced podocyte injury and renal fibrosis associated with activation of the SDF-1α/CXCR4 axis [68]. In the present study, the IR animals that underwent sitagliptin preconditioning had significantly lower expression of *Sdf1* and *Cxcr4* as well as lower receptor concentration than animals subjected to IR procedure without preconditioning. This observation is in line with an unfavorable role of SDF-1α/CXCR4 signaling in the liver [67], kidney [64,68], heart [65], or PC12 cells [66]. However, it opposes the response of endothelial [69] and progenitor endothelial cells to sitagliptin [70], in which the drug induced healing by employing SDF-1α/CXCR4 signaling to increase the proliferative, migratory, and angiogenic potential of cells. In fact, the role of the SDF-1α/CXCR4 axis in inflammatory diseases is rather ambiguous, as indicated by both beneficial and detrimental consequences of pathway inhibition [71].

## 4. Materials and Methods

### 4.1. Experimental Setting

The current study uses biobanked livers (stored at −80 °C), collected during the original experiment [15,16], which was conducted as briefly described below. The experiment was carried out on healthy animals to eliminate the harmful impact of diabetes on IR injury.

#### 4.1.1. Animals

Male Wistar rats, 2–3 months old, were housed under standard conditions (12:12 h day/night cycle, stable temperature of 19–21 °C, humidity of 45–60%, and continuous ventilation) with free access to standard food and water.

#### 4.1.2. Chemicals

Sitagliptin (Januvia—tablets 100 mg) was purchased from MSD (Warsaw, Poland), heparin (Heparinum WZF—ampoules 25,000 U/5 mL) from Polfa Warszawa (Warsaw, Poland), ketamine hydrochloride (Bio-ketan) from Vetoquinol Biowet (Gorzów Wlkp, Poland), medetomidine hydrochloride (Domitor, ampoules 1 mg/mL) from Orion Pharma (Warsaw, Poland), butorphanol tartrate (Morphasol, ampoules 4 mg/mL) from aniMedica GmbH (Frankfurt am Main, Germany), Ringer solution from Polfa Lublin S.A. (Lublin, Poland), and solution of 0.9% sodium chloride was obtained from Polpharma S.A. (Starogard Gdański, Poland).

#### 4.1.3. The IR Procedure

After a handling period of 2–3 weeks, the rats were randomly divided into four groups. Two groups were sham-operated: the group without drug delivery (controls; *n* = 9) and the group with sitagliptin (5 mg/kg p.o.) administered intragastrically two weeks prior to surgery once a day (sitagliptin, *n* = 8). The remaining two groups were subjected to the IR procedure: one group without prior drug administration (IR, *n* = 9) and one group where sitagliptin was administered in the same scheme as in the sitagliptin group (SIR, *n* = 10).

Before laparotomy, animals were anesthetized by the intramuscular administration of ketamine hydrochloride (7 mg/kg), medetomidine hydrochloride (0.1 mg/kg), and butorphanol tartrate (2 mg/kg). Ischemia of 70% of the liver (intermediate lobe and left lateral lobe) was induced as described previously [15,16] by placing a microvascular clamp over the portal vein and hepatic artery. After 60 min of ischemia, the microclips were removed, allowing reperfusion for 24 h. Animals in the non-ischemic groups underwent a sham surgery in which the blood vessels were isolated but not clamped. At the end of the experiment, the ischemic liver lobes were isolated and the fragments placed in a solution of RNAlater (Qiagen, Hilden, Germany) or frozen and stored at −80 °C for metabolomic and immunoenzymatic analyses.

During reperfusion, the activity of aminotransferases, as markers of hepatocyte injury, was determined in rat sera by a certified laboratory using commercially available enzymatic methods and, after the surgical procedure, a histological evaluation was performed under a light microscope [15,16].

### 4.2. Analytical Methods

#### 4.2.1. Metabolomic Analysis of Eicosanoids

##### Chemicals and Reagents

Methanol, acetonitrile (ACN), ethyl acetate, water, formic acid (FA) were acquired from Merck Millipore (Warsaw, Poland). Standards of Thromboxane B2, Leukotriene B4, Prostaglandin D2, Prostaglandin E2, 6-keto Prostaglandin F1α, Prostaglandin F2α, 15-deoxy-Δ12,14-Prostaglandin J2, 13,14-dihydro Prostaglandin E1, and their isotope-labeled standards were procured from Cayman Chemical Company (Ann Arbor, MI, USA).

##### Sample Preparation

Tissue samples (~0.5 g) were homogenized using ceramic beads in 1 mL of LC-MS-grade water in Bead Ruptor Elite homogenizer (Omni International, Kennesaw, GA, USA). Aliquotes of 100 µL of homogenates were mixed with 10 µL of internal standard and 20 μL of 0.2% FA in water. Samples were deproteinized and extracted with an ACN mixture with ethyl acetate. The obtained supernatants were evaporated to dryness and re-dissolved before analysis in 20% ACN in water.

##### LC-MS Analysis

LC-MS data were obtained using Acquity UPLC system (Waters, Milford, MA, USA), equipped with a single quadrupole-time of flight mass analyzer and an electrospray (ESI) ion source (Xevo G2 Q-TOF MS from Waters). Spectra were obtained in negative ionization mode with the following MS parameters: the sprayer voltage and the desolvation temperature were set at 2.0 kV and 450 °C, respectively. All scans were carried out in an MS/MS QTOF scan mode. Data acquisition and calculations were performed with MassLynx and QuanLynx software (Waters), respectively.

Analytes were separated using Acquity UPLC BEH Shield C18 1.7 µm chromatographic column (100 × 2.1 mm, 1.70 µm) from Waters with a linear gradient from 30% to 95% of mobile phase B in 7.2 min with a total flow rate of 250 µL/min. As mobile phases, 0.1% FA in water (A) and 0.1% FA in ACN (B) were used.

#### 4.2.2. Immunoassays

Rat livers (~0.5 g) were homogenized using ceramic lysing matrix beads in the Bead Ruptor Elite bead mill homogenizer (Omni International) with 0.5 mL of PBS buffer pH 7.4 (137 mM NaCl, 2.7 mM KCl, 10 mM Na_2_HPO_4_, and 1.8 mM KH_2_PO_4_) with 1 mM PMSF. Obtained homogenates were centrifuged (12,000× *g*, 10 min, 4 °C), and supernatants were collected, aliquoted, and stored at −80 °C until analysis.

Prior to analysis, tissue homogenates were clarified by centrifugation at 10,000× *g*, 10 min, 4 °C.

The concentrations of GLP-1 (glucagon-like peptide-1) and VIP (vasoactive intestinal peptide) were determined using RayBio^®^ Rat Enzyme Immunoassay Kits (#EIAR-GLP1 and #EIAR-VIP, respectively) from RayBiotech Life (Peachtree Corners, GA, USA) according to the manufacturer’s instructions. Samples were diluted at 1:50 and tested in duplicates. Standard curves were drawn using 5-parameter logistic (PL) regression.

The C-X-C chemokine receptor type 4 (CXCR4) and C-X-C motif chemokine 12 (CXCL12/SDF1α) were determined using, respectively, Nori^®^ Rat CXCR4 and Nori^®^ Rat CXCL12 ELISA Kits (Genorise Scientific Inc., Glen Mills, PA, USA) following manufacturer’s instructions. Samples were diluted 1:1 in Assay Buffer and tested in duplicates. The standard curves were calculated using a computer-generated 4-parameter logistic (PL) regression.

#### 4.2.3. Protein Determination

Protein concentration was determined using Coomassie Plus (Bradford) Assay Kit (Thermo Scientific, Waltham, MA, USA) against the BSA standard curve according to the manufacturer’s instructions. Samples were diluted in PBS and tested in duplicates.

Concentrations of eicosanoids and peptides/proteins were adjusted to protein content in the sample and expressed per mg of protein.

#### 4.2.4. Transcriptional Analysis

A cDNA library has been prepared from isolated RNA as described in the original study [15,16] and used for the present investigation.

All qPCR reactions were conducted in triplicates using the CFX96 platform (Biorad, Hercules, CA, USA) under standardized thermal cycling conditions: activation of the polymerase for 110 s at 95 °C followed by 40 cycles of denaturation (95 °C for 5 s) and annealing and synthesis (61.4 °C for 5 s). Melting curve analysis (60–95 °C, reading every 0.5 °C) was conducted to confirm the specificity of the reaction product. The qPCR mixture consisted of 2 µL of diluted 1:5 cDNA, 10 µL of 2× SsoFast EvaGreen^®^ Supermix (BioRad), 1 µL of each 10 nM forward and reverse target-specific primers, filled with water up to 20 µL. Primers were synthesized by Genomed (Warsaw, Poland) (Table 4). The relative gene expression was calculated as follows: geometric mean of all Cq values was subtracted from individual sample Cq (ΔCq), linearized by 2^ΔCq^ conversion, and normalized to *Gapdh* expression. The resulting values are referred to as normalized relative quantities (NRQ) [72].

#### 4.2.5. Inflammatory Mediators and Markers of Oxidative, Nitrosative, and Halogenative Stress

Data on inflammatory mediators and markers of oxidative and halogenative stress were retrieved from our earlier published studies [16,17] for the purpose of correlation analysis.

### 4.3. Statistical Analysis

Data were analyzed using MedCalc^®^ Statistical Software version 20.014 (MedCalc Software Ltd., Ostend, Belgium). Normality of data distribution and homogeneity of variances was established with Kolmogorov–Smirnov and Leven tests, respectively. Between-group comparisons were conducted using the Kruskal–Wallis H test with Conover post-hoc test, and results are presented as medians with interquartile range (IQR). Correlation analysis was conducted using Pearson correlation on log-transformed data, if appropriate. Multiple linear regression, a stepwise method, was applied to discern independent predictors of explained variables with explanatory variables entered into the model if *p* < 0.05 and removed if *p* > 0.1. The VIF (variance inflation factor) was calculated for each predictor variable to diagnose multilinearity. The repeated-measures ANOVA was conducted to discern IR and sitagliptin effect on the dynamics of liver enzymes activities. All calculated probabilities were two-tailed. The *p* values ≤ 0.05 were considered statistically significant.

## 5. Conclusions

In the present study, we demonstrated that hepatic IR is associated with an increase in tissue concentrations of 6-ketoPGF_1α_ (PGI_2_), PGE_2_, and 13,14-dihydro-PGE_1_ and with an elevated 6-ketoPGF_1α_ (PGI_2_)/TXB_2_ (TXA_2_) ratio, which was abrogated by pretreatment of IR animals with sitagliptin. As the drug has an opposite effect regarding 6-ketoPGF_1α_ (PGI_2_) and 6-ketoPGF_1α_ (PGI_2_)/TXB_2_ (TXA_2_) ratio in control sham-operated animals, it might indicate that the biological effect of sitagliptin is context-dependent. We also showed that sitagliptin upregulated PGD_2_ and 15-deoxy-12,14-PGJ_2_, which seems to be beneficial in the light of the recently reported hepatoprotective effect of the prostaglandin in the course of ischemia and reperfusion.

In order to shed some light on molecular mechanisms underlying favorable outcomes associated with sitagliptin pretreatment, we investigated drug effects on DPP4 substrates potentially relevant for liver protection during IR injury and on their receptors. We found that the IR upregulated *Cxcr4*/CXCR4 and downregulated VIP and *Vpac2* but not in animals treated with sitagliptin.

Taken together, our results indicate that beneficial effects of sitagliptin during hepatic IR injury might be mediated by drug-induced normalization of proinflammatory prostanoids and upregulation of PGD_2_ and by concomitant downregulation of SDF-1α/CXCR4 and reinstating VIP/VCAP2 signaling.

## Figures and Tables

**Table 1:** Liver profile of eicosanoids—effect of IR injury and sitagliptin.

Metabolite	Median Prostanoid Concentration (pg/mg) (IQR)				*p*
	Control, *n* = 9	IR, *n* = 9	SIR, *n* = 10	Sitagliptin, *n* = 8	
6-ketoPGF_1α_ (PGI_2_)	68.0 (72.5) ^2,4^	247.5 (70.5) ^1,3^	121.8 (48.3) ^2,4^	211 (141.5) ^1,3^	0.001
PGE_2_	154.7 (127) ^2^	263.3 (74.4) ^1,3^	184.7 (39) ^2^	225.4 (160)	0.050
PGF_2_	58.7 (35.5)	55.1 (17.7)	57.4 (27.7)	62.4 (25.8)	0.962
PGD_2_	571 (849) ^4^	1142 (434) ^4^	1180 (493) ^4^	1555 (144) ^1,2,3^	0.002
15-deoxy-12,14-PGJ_2_ (PGD_2_)	1.04 (1.47) ^3,4^	1.15 (1.87) ^3,4^	2.88 (2.48) ^1,2^	3.2 (1.23) ^1,2^	0.004
13,14-dihydro-PGE_1_ (PGE_1_)	0.066 (0.11) ^2^	0.15 (0.12) ^1,3^	0.053 (0.06) ^2^	0.082 (0.10)	0.043
TXB_2_ (TXA_2_)	6.19 (4.42)	3.6 (2.85)	3.44 (5.0)	4.61 (3.0)	0.559
LTB_4_ (LTA_4_)	41.9 (102)	136.2 (66.9)	90.6 (124.1)	124.1 (102.5)	0.495
6-ketoPGF_1α_ (PGI_2_)/TXB_2_ (TXA_2_)	15.7 (34.4) ^2,4^	75.2 (72.9) ^1,3^	21.6 (45.4) ^2^	46.3 (39.2) ^1^	0.015

Data were analyzed using Kruskal–Wallis *H* test with Conover post-hoc analysis and are presented as medians with IQR. ^1^, significantly different from the control group; ^2^, significantly different from IR group; ^3^, significantly different from SIR group; ^4^, significantly different from sitagliptin group. IQR, interquartile range; *n*, number of observations; IR, ischemia-reperfusion; SIR, sitagliptin pretreatment, and ischemia-reperfusion. Metabolite precursors are given in brackets.

**Table 2:** Regression models explaining variability in the liver concentration of eicosanoids.

Dependent Variable	Explanator Variables	Regression Coefficient (*β*), *p*	*r*_p_	VIF	R^2^; ANOVA
6-ketoPGF_1α_ (PGI_2_)	(Constant)	100.4			R^2^ = 0.832; *F* = 19.1, *p* < 0.0001
	13,14-dihydro-PGE_1_	321.3, *p* = 0.003	0.53	1.07	
	PGE_2_	0.26, *p* = 0.028	0.41	1.63	
	*Cxcr4* (log)	56.7, *p* = 0.022	0.42	1.32	
	3-NT	1.56, *p* < 0.001	0.64	1.18	
	*Nox4* (log)	−22.0, *p* = 0.055	−0.36	1.64	
	VIP	−0.73, *p* = 0.025	−0.42	1.39	
	IL-1β	0.07, *p* = 0.002	0.55	1.21	
PGE_2_	(Constant)	23.11			R^2^ = 0.744; *F* = 30.0, *p* < 0.0001
	6-ketoPGF_1α_ (PGI_2_)	0.33, *p* < 0.001	0.57	1.17	
	PGF_2_	1.57, *p* < 0.001	0.63	1.26	
	LTB_4_	0.33, *p* = 0.006	0.47	1.33	
PGF_2_	(Constant)	3.10			R^2^ = 0.613; *F* = 26.1, *p* < 0.0001
	PGE_2_	0.18, *p* < 0.001	0.69	1.05	
	TXB_2_ (log)	33.9, *p* = 0.002	0.52	1.05	
PGD_2_	(Constant)	126.7			R^2^ = 0.729; *F* = 20.8, *p* < 0.0001
	15-deoxy-12,14-PGJ_2_	145.5, *p* < 0.001	0.59	1.10	
	PGE_2_	2.27, *p* = 0.007	0.46	1.40	
	SDF1α	4.86, *p* = 0.026	0.39	1.50	
	Sitagliptin	405, *p* = 0.005	0.48	1.19	
15-deoxy-12,14-PGJ_2_	(Constant)	0.20			R^2^ = 0.453; *F* = 13.7, *p* < 0.001
	PGD_2_	0.002, *p* < 0.001	0.60	1.00	
	Sitagliptin	1.25, *p* = 0.006	0.45	1.00	
13,14-dihydro-PGE_1_	(Constant)	0.08			R^2^ = 0.161; *F* = 6.54, *p* = 0.015
	IR	0.07, *p* = 0.015	0.40	1.00	
TXB_2_ (log)	(Constant)	0.24			R^2^ = 0.279; *F* = 12.8, *p* = 0.001
	PGF_2_	0.01, *p* = 0.001	0.53	1.00	
LTB_4_	(Constant)	−42.2			R^2^ = 0.624; *F* = 17.7, *p* < 0.0001
	15-deoxy-12,14-PGJ_2_	17.6, *p* = 0.002	0.50	1.02	
	PGE_2_	0.42, *p* = 0.001	0.53	1.40	
	SDF1α	0.72, *p* = 0.027	0.38	1.42	

Data were analyzed using the stepwise method of linear multivariate regression. Variables found significantly associated with dependent variables were entered into the analysis. Results are presented as regression coefficients *β* together with corresponding *p*-value and partial correlation coefficient (*r*_p_) for each explanatory variable retained in the regression model and as the model’s coefficient of determination (R^2^) together with ANOVA results (*F* statistics and *p*-value). VIF, variable inflation factor.

**Table 3:** Regression models explaining variability in the expression and/or concentrations of DPP4 ligands and their receptors.

Dependent Variable	Explanatory Variables	Regression Coefficient (*β*), *p*	*r*_p_	VIF	R^2^; ANOVA
CXCR4	(Constant)	48.1			R^2^ = 0.390; *F* = 10.2, *p* < 0.001
	6-ketoPGF_1α_ (PGI_2_)	0.07, *p* = 0.034	0.36	1.31	
	IR	15.8, *p* = 0.027	0.38	1.31	
*Cxcr4* (log)	(Constant)	−0.11			R^2^ = 0.376; *F* = 19.9, *p* < 0.0001
	IR	0.50, *p* < 0.001	0.61	1.00	
*Sdf1* (log)	(Constant)	0.002			R^2^ = 0.817; *F* = 46.1, *p* < 0.0001
	*Nox4* (log)	0.16, *p* < 0.001	0.60	1.13	
	*Vpac1* (log)	0.40, *p* < 0.001	0.65	1.57	
	*Nampt* (log)	0.62, *p* < 0.001	0.62	1.41	
SDF1α	(Constant)	−12.8			R^2^ = 0.436; *F* = 12.7, *p* < 0.001
	LTB_4_	0.16, *p* = 0.028	0.37	1.54	
	PGD_2_	0.02, *p* = 0.030	0.37	1.54	
*Vpac1* (log)	(Constant)	0.003			R^2^ = 0.893; *F* = 85.8, *p* < 0.0001
	*Mdk* (log)	0.38, *p* < 0.001	0.82	1.27	
	*Nox2* (log)	−0.18, *p* = 0.043	−0.36	1.13	
	*Sdf1* (log)	0.62, *p* < 0.001	0.83	1.24	
*Vpac2* (log)	(Constant)	0.17			R^2^ = 0.231; *F* = 9.88, *p* = 0.004
	IR	−0.78, *p* = 0.004	−0.48	1.00	
VIP	(Constant)	200.0			R^2^ = 0.388; *F* = 6.55, *p* = 0.002
	PGE_2_	−0.09, *p* = 0.073	−0.32	1.13	
	3-BT	−1.95, *p* = 0.040	−0.36	1.01	
	IR	−23.1, *p* = 0.021	−0.40	1.12	
*Glp1r* (log)	(Constant)	−0.02			R^2^ = 0.829; *F* = 77.3, *p* < 0.0001
	*Nox1* (log)	1.02, *p* < 0.001	0.89	1.10	
	*Nox2* (log)	−0.67, *p* = 0.030	−0.37	1.10	
GLP1	(Constant)	49.9			R^2^ = 0.315; *F* = 7.37, *p* = 0.002
	IFNγ	−0.004, *p* =0.019	−0.40	1.00	
	IR ^1^	14.4, *p* =0.006	0.47	1.00	

Data were analyzed using the stepwise method of linear multivariate regression. Variables found significantly associated with dependent variables were entered into the analysis. Results are presented as regression coefficients *β* together with corresponding *p*-value and partial correlation coefficient (*r*_p_) for each explanatory variable retained in the regression model and as the model’s coefficient of determination (R^2^) together with ANOVA results (*F* statistics and *p*-value). IR, ischemia-reperfusion. ^1^, IR and SIR groups combined to analyze the effect of the IR component. VIF, variable inflation factor.

**Table 4:** Primers’ sequences.

Gene	Forward Sequence	Reverse Sequence
*Gapdh*	TGACTCTACCCACGG-CAAGTTCAA	ACGACATACTCAGCACCAG-CATCA
*Cxcr4*	GCTGGAGAGCGAGCATTG	TAGATGGTGGGCAGGAAGATCC
*Sdf1*	CTCAACACTCCAAACTGTGCCC	GTCCAGGTACTCTTGGATCCAC
*Pac1*	GGCTGTGCTGAGGCTCTATTTTG	AGGATGATGATGATGCCGATGA
*Vpac1*	GATGTGGGACAACCTCACCTG	TAACCATGAATGGGGGCAAAC
*Vpac2*	GGTGAGCAGCATCCACCCAG	TCACTAGTGCAGTTTTTGCTTA
*Glp1r*	GGGTATCTGGCTGCATAAGGACAAC	AAGGATGGCTGAAGCGATGAC

## Data Availability

Data sharing is not applicable.

## Supplementary Materials

The following are available online at www.mdpi.com/article/10.3390/ijms222313155/s1.
