# Supplementary material for: Modulation of Prostanoids Profile and Counter-Regulation of SDF-1α/CXCR4 and VIP/VPAC2 Expression by Sitagliptin in Non-Diabetic Rat Model of Hepatic Ischemia-Reperfusion Injury
Source: Int J Mol Sci. 2021 Dec 5;22(23):13155. doi: 10.3390/ijms222313155 (PMC8658172; doi:10.3390/ijms222313155)
Supplement: Supplementary file 1 [file ijms-22-13155-s001.zip › ijms-1468337-supplementary.pdf]

Supplementary Material

Modulation of Prostanoids Profile and Counter-Regulation of SDF-1α/CXCR4 and VIP/VPAC2 Expression by Sitagliptin in Non-Diabetic Rat Model of Hepatic Ischemia-Reperfusion Injury

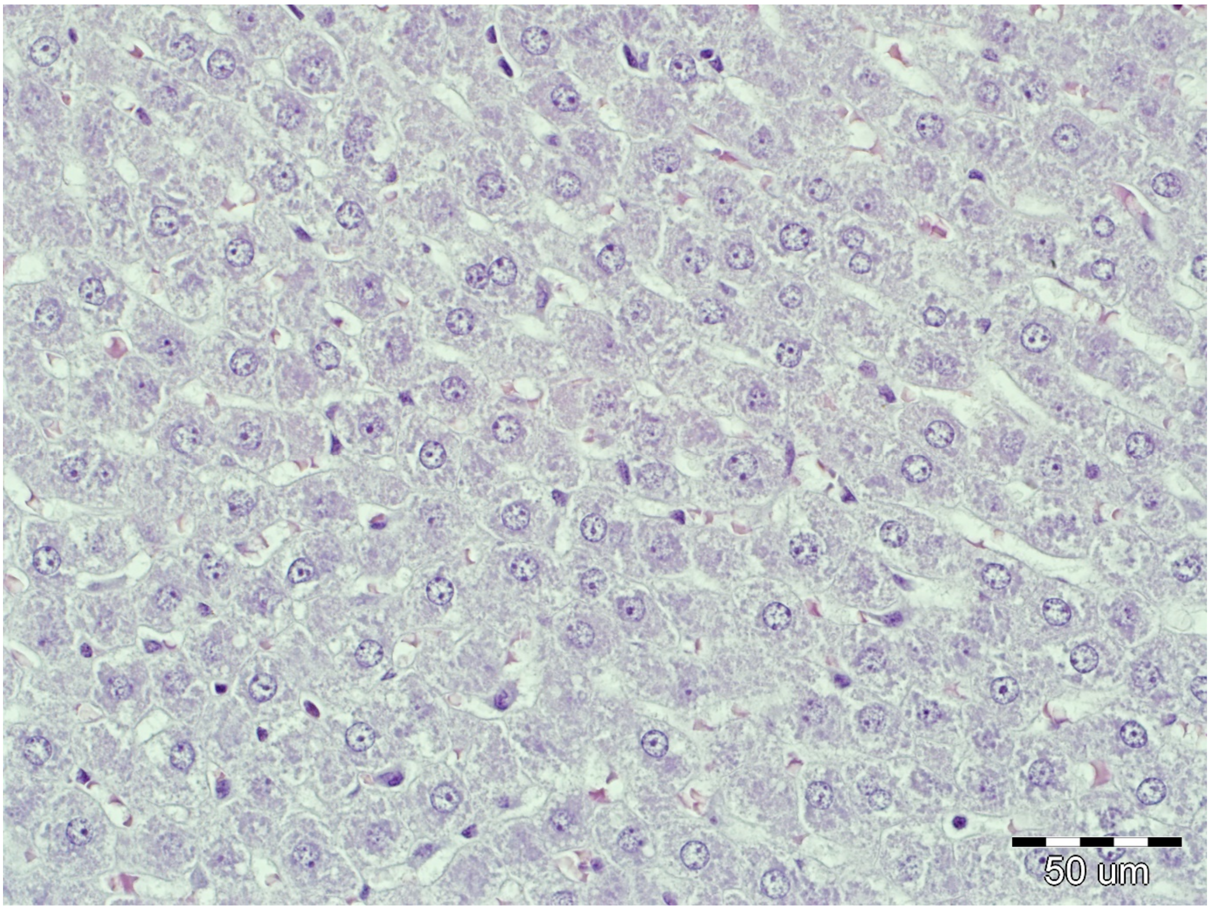

Figure S1. Histological findings validating the established rat model of hepatic ischemia-reperfusion injury—exemplary liver tissue from a control group animal (sham-operated, no pretreatment with sitagliptin). Slides were stained with hematoxylin and eosin and are presented at ×400 magnification with a reference bar corresponding with 50 μm.

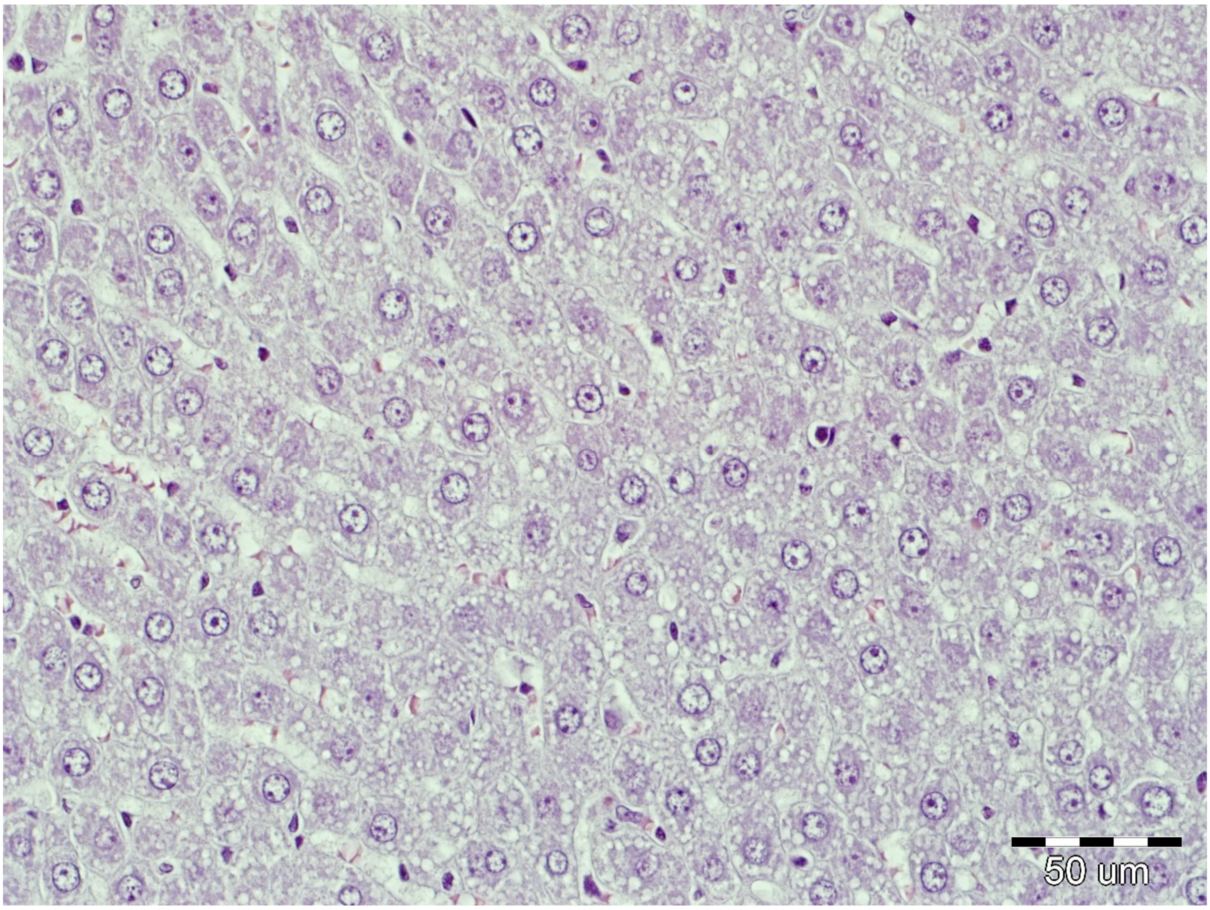

Figure S2. Histological findings validating the established rat model of hepatic ischemia-reperfusion injury—exemplary liver tissue from a sitagliptin group animal (sham-operated, pretreatment with sitagliptin). Slides were stained with hematoxylin and eosin and are presented at ×400 magnification with a reference bar corresponding with 50 μm.

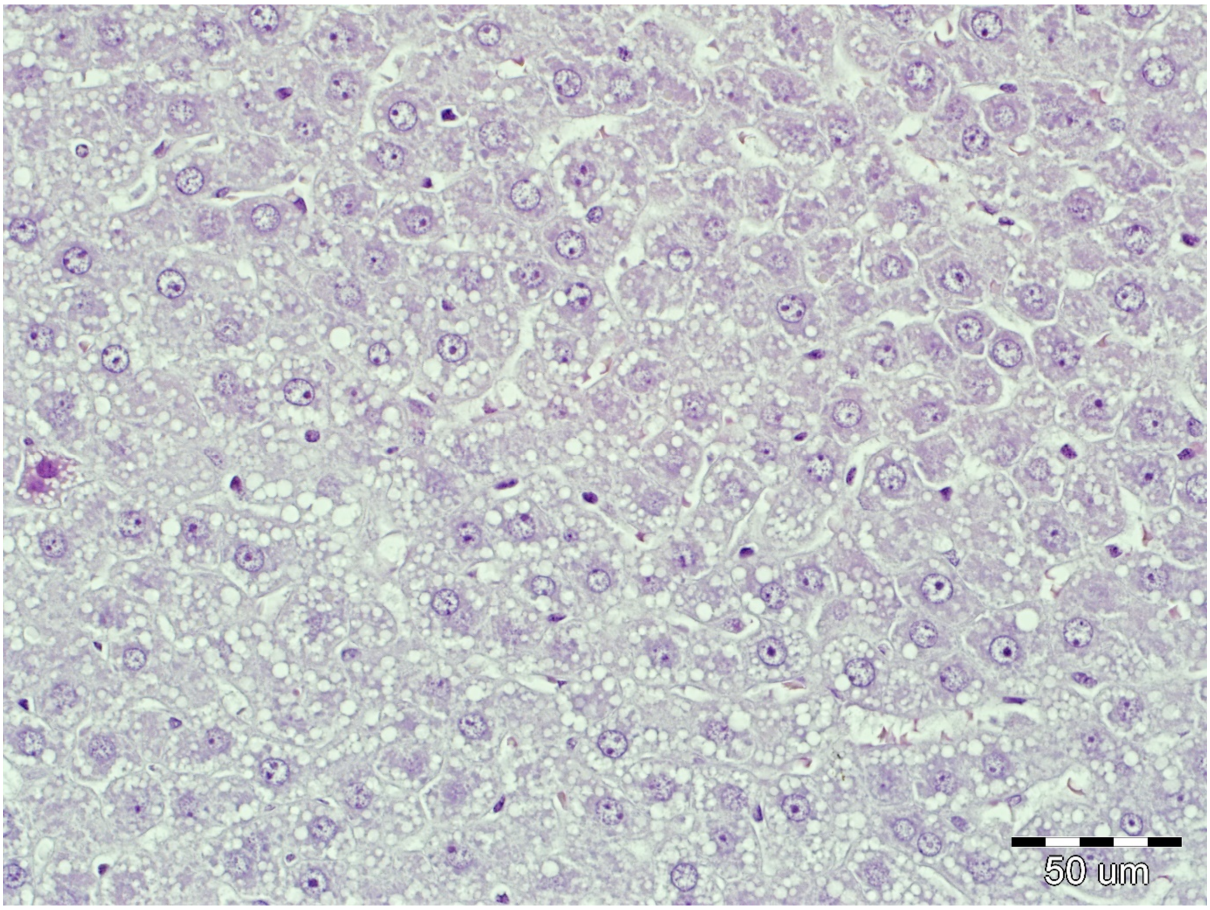

Figure S3. Histological findings validating the established rat model of hepatic ischemia-reperfusion injury—exemplary liver tissue from an IR group animal (subjected to ischemia-reperfusion, no pretreatment with sitagliptin). Slides were stained with hematoxylin and eosin and are presented at ×400 magnification with a reference bar corresponding with 50 μm.

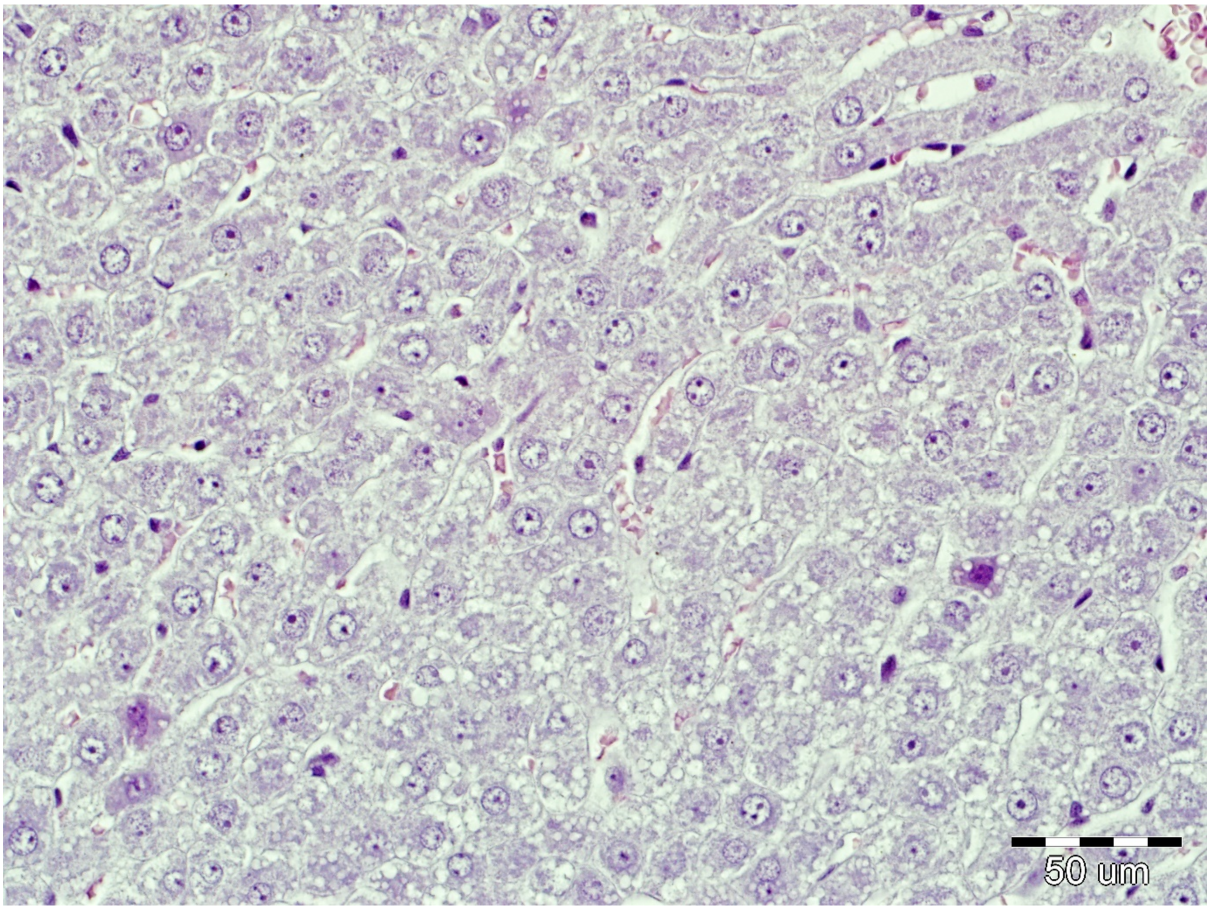

Figure S4. Histological findings validating the established rat model of hepatic ischemia-reperfusion injury—exemplary liver tissue from a SIR group animal (subjected to ischemia-reperfusion, pretreatment with sitagliptin). Slides were stained with hematoxylin and eosin and are presented at ×400 magnification with a reference bar corresponding with 50 μm.
